# Tissue Engineering of Ureteral Grafts: Preparation of Biocompatible Crosslinked Ureteral Scaffolds of Porcine Origin

**DOI:** 10.3389/fbioe.2015.00089

**Published:** 2015-06-23

**Authors:** Holger Koch, Niels Hammer, Susann Ossmann, Katrin Schierle, Ulrich Sack, Jörg Hofmann, Mike Wecks, Andreas Boldt

**Affiliations:** ^1^Translational Centre for Regenerative Medicine (TRM), University of Leipzig, Leipzig, Germany; ^2^Institute of Anatomy, Faculty of Medicine, University of Leipzig, Leipzig, Germany; ^3^Heart Center, Clinic for Cardiac Surgery, University of Leipzig, Leipzig, Germany; ^4^Institute of Pathology, University of Leipzig, Leipzig, Germany; ^5^Institute for Clinical Immunology, Faculty of Medicine, University of Leipzig, Leipzig, Germany; ^6^Institut für Nichtklassische Chemie e. V., Leipzig, Germany

**Keywords:** ureter, scaffold, decellualrization, tissue engineering, crosslinking

## Abstract

The surgical reconstruction of ureteric defects is often associated with post-operative complications and requires additional medical care. Decellularized ureters originating from porcine donors could represent an alternative therapy. Our aim was to investigate the possibility of manufacturing decellularized ureters, the characteristics of the extracellular matrix (ECM) and the biocompatibility of these grafts *in vitro/in vivo* after treatment with different crosslinking agents. To achieve these goals, native ureters were obtained from pigs and were decellularized. The success of decellularization and the ECM composition were characterized by (immuno)histological staining methods and a DNA-assay. *In vitro*: scaffolds were crosslinked either with carbodiimide (CDI), genipin (GP), glutaraldehyde, left chemically untreated or were lyophilized. Scaffolds in each group were reseeded with Caco2, LS48, 3T3 cells, or native rat smooth muscle cells (SMC). After 2 weeks, the number of ingrown cells was quantified. *In vivo*: crosslinked scaffolds were implanted subcutaneously into rats and the type of infiltrating cells were determined after 1, 9, and 30 days. After decellularization, scaffold morphology and composition of ECM were maintained, all cellular components were removed, DNA destroyed and strongly reduced. *In vitro*: GP and CDI scaffolds revealed a higher number of ingrown 3T3 and SMC cells as compared to untreated scaffolds. *In vivo*: at day 30, implants were predominantly infiltrated by fibroblasts and M2 anti-inflammatory macrophages. A maximum of MMP3 was observed in the CDI group at day 30. TIMP1 was below the detection limit. In this study, we demonstrated the potential of decellularization to create biocompatible porcine ureteric grafts, whereas a CDI-crosslink may facilitate the remodeling process. The use of decellularized ureteric grafts may represent a novel therapeutic method in reconstruction of ureteric defects.

## Introduction

The surgical removal of tissues or organs is an aggressive therapeutic strategy to treat innate or acquired pathologies of the urinary tract. Surgical removal will present the patient and surgeon with challenges in the reconstruction of the excretory function of the urinary tract. In the case of extensive ureteral resection, surgical reconstruction by means of end-to-end anastomoses remains a major issue. Various surgical techniques were employed such as the psoas hitch (Warwick and Worth, 1969), the Boari flap (Boari, 1894) and the downward mobilization of the respective kidney (Sullivan et al., 1982), as well as the replacement of the ureters with ileal or bladder tissue or even nephrectomy (del Pizzo et al., 1998; Corvin et al., 2004; Schlote et al., 2004; Wolff et al., 2011; Takeuchi et al., 2014). These surgical techniques were frequently accompanied by serious complications, ranging from mucus formation, stenosis, and infection to renal failure (Corvin et al., 2004; Schlote et al., 2004; Wolff et al., 2011). To circumvent these complications, tissue-engineered scaffolds may be an alternative in the surgical reconstruction of the urinary tract.

Recently, various types of synthetic or biologic scaffolds (Ulm and Krauss, 1960; Block et al., 1977) and hydrogels (Dahms et al., 1997) were introduced in ureteral reconstruction, including decellular scaffolds. Synthetic scaffolds often have a lack in biocompatibility, peristaltic motion, and incrustation. Decellular scaffolds have several advantages over other implants. They can be obtained easily from various species. The cellular components can be removed from the tissues using a broad range of methods (Gilbert et al., 2006; Koch et al., 2012), resulting in decellular extracellular matrix (ECM). Mostly, the structural ECM proteins remain intact three dimensionally, facilitating host cell ingrowth (Badylak, 2004; Koch et al., 2012). Furthermore, antigenic binding sites were strongly reduced due to the removal of DNA and other cellular components. On the other hand, inflammatory reactions within the tissue and degradation cannot entirely be excluded. Inflammatory reactions are likely to be caused by the ECM proteins, providing co-stimulatory signals to immune cells (Lider et al., 1995; Tanemura et al., 2000; Allman et al., 2001, 2002; Badylak, 2004; Konakci et al., 2005; Morwood and Nicholson, 2006; Adair-Kirk and Senior, 2008; Badylak and Gilbert, 2008; Koch et al., 2012). The intensity of the inflammatory reaction and the subsequent degradation and remodeling of decellular scaffolds is strongly tissue dependent and also influenced by the chemical treatment (Badylak, 2004; Gilbert et al., 2006; Valentin et al., 2006; Badylak and Gilbert, 2008; Koch et al., 2012). Though preliminary results with decellular matrices yielded promising results concerning the ingrowth of urothelium, of smooth muscle cells (SMC) and nerve fibers (Dahms et al., 1997). Therefore, decellular scaffolds have to meet two goals. First, ECM scaffolds should maintain their mechanical properties, providing an adequate matrix that allows the cell ingrowth *in vivo*. Second, moderate scaffold degradation is necessary to allow for tissue remodeling. To meet these goals, chemical crosslinking agents are applied (Koch et al., 2012).

The goals of the given study were as follows: (A) to characterize tissue-engineered decellular porcine ureteral scaffolds and (B) to determine the influence of crosslinking agents on the morphological matrix properties and cell ingrowth *in vitro*. Furthermore, we aimed (C) to investigate whether decellular ureters might provoke inflammatory responses or rejection reactions. Another goal of the given study was to evaluate a method to manufacture scaffolds suitable for storage by means of lyophilization.

We addressed the following hypotheses:

1. Porcine ureters can be decellularized successfully, thus preserving ECM composition.
2. Tissue-engineered decellular ureteral scaffolds can be reseeded with different cells *in vitro* and do not provoke rejection reactions *in vivo*.
3. Inflammatory reactions and degradation rate might be influenced by different crosslinking agents *in vivo*.

## Materials and Methods

### Porcine ureteral scaffolds

All experiments were performed with ureters from pigs (*Deutsche Landrasse*, 25–65 kg). The methods used in the present study were similar in some aspects to those of earlier published work (Koch et al., 2012) and complemented by DNA- (2.2), SDS- (2.3), and reseeding-assays (2.6). The organs were obtained under sterile conditions and were stored at 4°C in a 0.9% NaCl solution. For decellularization, ureters were cut into pieces of 8 cm length. The tunica adventitia was removed mechanically. Ureters were then placed in a 1% sodium dodecyl sulfate solution (SDS; Roth, Karlsruhe, Germany) for 7 days. Afterwards, the scaffolds were washed in distilled water for 7 days, sterilized by gamma radiation (25 kGy from a ^60^Co source) and stored in PBS at 4°C for a maximum of 4 weeks. SDS solution and distilled water were changed daily.

### DNA quantification and qualitative fragment analysis

The isolation and quantification of DNA in the decellular tissue scaffolds was performed using the protocol of Qiagen (DNeasy™, Hilden, Germany) and as previously described (Koch et al., 2012). In brief, decellular ureteral scaffolds were cut into small cross-sectional pieces of 25 mg each and proteinase K (Qiagen, in lysis buffer) was added. Following incubation for 12 h in a shaking water bath (56°C), the DNA was purified and measured spectrophotometrically using a Nanodrop Spectrophotometer (Peqlab, Erlangen, Germany). The DNA content of matrix scaffolds undergoing enzymatic digestion with DNase (200 μg/ml; Sigma, Deisenhofen, Germany; 12 h at 37°C) was compared to the scaffolds without enzymatic digestion (both *n* = 9). Native ureters with the same origin served as positive control (*n* = 9). For qualitative fragment length analysis, 5–12 μg of total DNA was electrophoretically separated in a 1.5% agarose gel (50 min, 120 V). After the run, the gel was documented by light exposure in the FastGeneGelPic LED Bos (Nippon Genetics Europe, Dueren, Germany).

### SDS assay

The determination of residual SDS was performed using an anionic surfactants test kit (Nanocolor^®^, Macherey-Nagel GmbH & Co. KG, Düren, Germany). Dried decellular ureteral scaffolds were cut into small cross-sectional pieces of 100 mg and were homogenized using liquid nitrogen. In addition, scaffold powder was dissolved in distilled water (4 ml) and added to test tubes containing 4 ml chloroform and 2 ml methanol (5%). Furthermore, 0.5 ml of a methylene blue solution (1%) was added and mixed intensively for 1 min. After incubation time of 10 min, the absorption of the chloroform-containing phase was measured spectrophotometrically at the absorption maximum of 660 nm. For the calculation of residual SDS concentrations within scaffold pieces before and after washing (*n* = 12), a calibration curve was prepared.

### Lyophilization of ureteral scaffolds

For lyophilization, the sterilized scaffolds were placed in a plastic bowl filled with distilled water at room temperature. The bowls were immersed in a vessel filled with liquid nitrogen until distilled water containing the scaffolds was frozen. The frozen scaffolds were then placed in a freeze-dryer (Alpha 1–4 LDC-1, Martin Christ Gefriertrocknungsanlagen GmbH, Osterode, Germany) and scaffolds were lyophilized applying a constant vacuum (0.05 mbar, RZ-5, Vacuubrand, Wertheim, Germany) in a range from −40 to 0°C.

### Crosslinking of ureteral scaffolds

Scaffold-crosslinking with different agents was performed as previously described (Koch et al., 2012). In brief, for genipin (GP) crosslinking, the scaffolds were incubated in a 0.33% GP/ethanol solution (Alexis, Lausen, Switzerland) for 3 days at 37°C. Then the scaffolds were removed and rinsed in 75% ethanol for 2 h and in PBS for 3 days (Sung et al., 1999b; Liang et al., 2004; Mantovani et al., 2005; Koch et al., 2012). For carbodiimide (CDI) crosslinking circular pieces of ureter scaffolds (3 mm thickness) were immersed in 2-(N-morpholino) ethanesulfonic acid buffer (MES buffer; 0.2 M, pH 5.0; Sigma, Munich, Germany). After 1 h, the MES buffer was discarded and the scaffolds were incubated in a solution consisting of MES buffer (0.2 M, pH 5.0), N-hydroxysuccinimide (NHS; 0.12 M), and N-(3-dimethylaminopropyl)-N-ethylcarbodiimide (EDC; 0.3 M). After 16 h, the scaffolds were removed and rinsed in MES buffer for 24 h and in PBS for at least 24 h (Cao and Xu, 2008; Everaerts et al., 2008; Koch et al., 2012). For glutaraldehyde (GA) crosslinking, the scaffolds were immersed in 0.625% glutaraldehyde/distilled water (Sigma) for 3 days at 37°C. Subsequently, the scaffolds were removed and washed in PBS for 3 days (Chang et al., 2005; Koch et al., 2012).

### Reseeding decellular ureteral scaffolds

Sterilized decellular scaffold pieces of 1 cm, which were either crosslinked, chemically untreated or lyophilized were placed in tubes (15 ml, BD Falcon^®^, Becton, Dickinson and Company, Heidelberg, Germany) containing different cell lines (Caco2, LS48, 3T3) or native rat SMC. Scaffolds and cells were incubated (each 1.5–2.0 × 10^6^ cells/tube) on a rotator (Multi-RS 60, Biosan, Riga, Latvia) at room temperature for 4 h. Afterwards, scaffolds were placed in well plates and incubated under constant conditions (37°C, 5% CO_2_) for 2 weeks (*n* = 7–9). After incubation, scaffold pieces were fixed in 4% paraformaldehyde solution and embedded in paraffin for further histological investigations.

### Subcutaneous rat model

110 Sprague-Dawley rats were grouped according to scaffold crosslinking: GA, GP, CDI, BP, untreated group, and sham group (each treatment group: *n* = 18; sham group: *n* = 20; for more details please refer to Table 1). One scaffold was implanted subcutaneously into each of the individuals of the respective groups. Animals of the sham group underwent the same surgical procedure but received no implant. The subcutaneous implantation of scaffold pieces was performed as previously described (Koch et al., 2012). In brief, animals were anesthetized with 5% isoflurane (Baxter Deutschland GmbH, Unterschleissheim, Germany) in a N_2_/O_2_ gas mixture. After anesthetization, the concentration of isoflurane was reduced to 2% isoflurane in a N_2_/O_2_ gas mixture to maintain anesthesia. Circular pieces of ureteral scaffolds (3 mm thickness, 1 cm diameter) were implanted into one of the subcutaneous back pockets of rats (1 cm length). The wound was sewn with two stitches. Finally, the concentration of isoflurane was reduced to allow rats to recover from anesthesia. All surgical interventions were performed under sterile conditions. Postoperatively, rats were given carprofen (5 mg/kg s.c., Pfizer, Berlin, Germany) for 3 days. After a follow-up of one, nine, or 30 days, the animals were again narcotized, euthanized, and the scaffolds were explanted for further histological investigation. Therefore, the scaffolds were immersed in 4% paraformaldehyde solution and embedded in paraffin. All procedures were approved by the committee of Animal Care and Use of the relevant local governmental body (TVV15/10) in accordance with the Animal Welfare Act. Every effort was made to minimize the number of animals used.

**Table 1:** **Group composition with type and crosslinking of scaffolds**.

Group	*n*	Type of scaffold	Scaffold crosslinking
Untreated	18	Decellularized, sterilized ureter scaffold	Untreated
GA	18	Decellularized, sterilized ureter scaffold	Glutaraldehyde
GP	18	Decellularized, sterilized ureter scaffold	Geripin
CDI	18	Decellularized, sterilized ureter scaffold	Carbodiimide
BP (control)	18	Decellularized, sterilized bovine pericard scaffold	Glutaraldehyde (bovine pericardium; St. Jude, USA)
Sham (negative control)	20	–	–

### Histology

Following decellularization and explantation at days 1, 9, and 30 post implantation, the scaffolds were fixed in paraformaldehyde solution. Representative areas were embedded in paraffin wax, cut into slices (5 μm thickness), and routinely stained with Hematoxylin–Eosin and Azan (Boldt et al., 2006; Koch et al., 2012). Using these slices, matrix morphology of decellular porcine scaffolds was compared to that of native ureters using light microscopy. Furthermore, the degree of scaffold infiltration by giant cells, granulocytes, capillaries, collagen fibers, lymphocytes, and fibroblasts at days 1, 9, and 30 post implantation was analyzed using these slices.

### Immunohistochemistry

Immunohistochemical analysis of decellular ureters was performed as previously described (Koch et al., 2012). Briefly, formalin-fixed, paraffin-embedded ureteral tissue sections of 5 μm thickness were deparaffinized. Subsequently, the slices were heated in 50 mM Tris buffered saline solution at 95°C for 15 min. After cooling, the slides were incubated with proteinase K (250 μg/ml) for 10 min and washed in distilled water. Endogenous enzyme activity was blocked (10 min, DAKO staining kit; DAKO Deutschland GmbH, Hamburg, Germany) and the tissue slides were incubated with primary antibodies. The staining steps with anti-collagen I (Acris Antibodies, Herford, Germany), anti-collagen III (Acris Antibodies), anti-collagen IV (Acris Antibodies), anti-elastin (Acris Antibodies), and anti-fibronectin (Dianova, Berlin, Germany) were performed following the manufacturer’s instruction (Envision DAB Staining Kit, DAKO). Control experiments were carried out without primary antibodies. All antibodies were diluted 1:100 in PBS. In the stained slices, the ECM composition of decellular and native scaffolds was investigated under light microscopy. To investigate cellular infiltration in explanted scaffolds of the rat model, anti-CD68 and anti-CD163 (all Serotec, Oxford, UK) antibodies were used. All antibodies were diluted 1:50 in PBS. Control experiments were carried out without primary antibodies. To visualize the nuclei, all slices were counterstained with Mayer’s hemalaun solution. In stained slices, from each section the CD68 and CD163 positive cells as well as their nuclei were counted from each section in 5 × 6 random microscopic fields by two observers, blinded to the origin of the tissue and each other’s ratings (magnification × 1000). The data are represented as a ratio of CD-positive cells/nuclei [mean value ± (SEM)]. To investigate the degradation of the collagen in the scaffolds, metallopeptidase 3 (MMP3) and the metallopeptidase inhibitor 1 (TIMP1) levels were analyzed, using the respective antibodies: anti-MMP3 and anti-TIMP1 (Bioss Inc., Woburn, MA, USA). All antibodies were diluted 1:100 in PBS. Control experiments were carried out without primary antibodies.

### Statistics

The Shapiro–Wilk test was used to determine normal distribution of the data, except immunohistochemistry data. The statistical evaluation of the immunohistochemistry including CD68 + or CD163 + cells was performed using Kruskal–Wallis one-way analysis of variance (ANOVA) on ranks with *post hoc* Tukey test. Statistical evaluation of the reseeding procedure, the effectiveness of the washing of the decellular scaffolds on residual SDS and the respective DNA concentration was performed with the one-way repeated measures (RM) ANOVA on ranks with *post hoc* Tukey test. *P*-values of *P* < 0.05 were considered statistically significant.

## Results

### Porcine ureteral scaffolds

**Figure 1:**
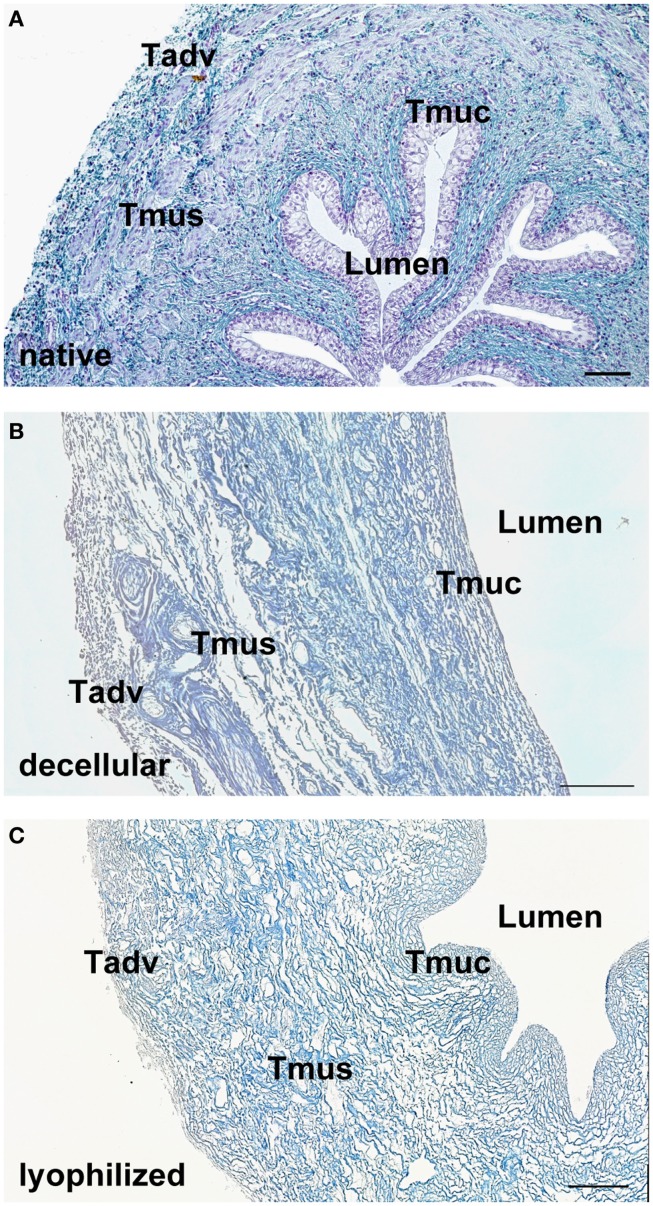
**Tissue morphology of native (A) and decellularized (B) porcine ureter**. Decellular ureteral scaffolds showed anatomically intact structures, ideal matrix geometry, and no remaining cellular structures. Lyophilization of decellular ureters showed no effects on anatomical structures **(C)**. Muscle cells, erythrocytes, and chromatin were stained red, connective tissue blue. Tadv, Tunica adventitia; Tmus, Tunica muscularis; Tmuc, Tunica mucosa. Bar = 100 μm.

**Figure 2:**
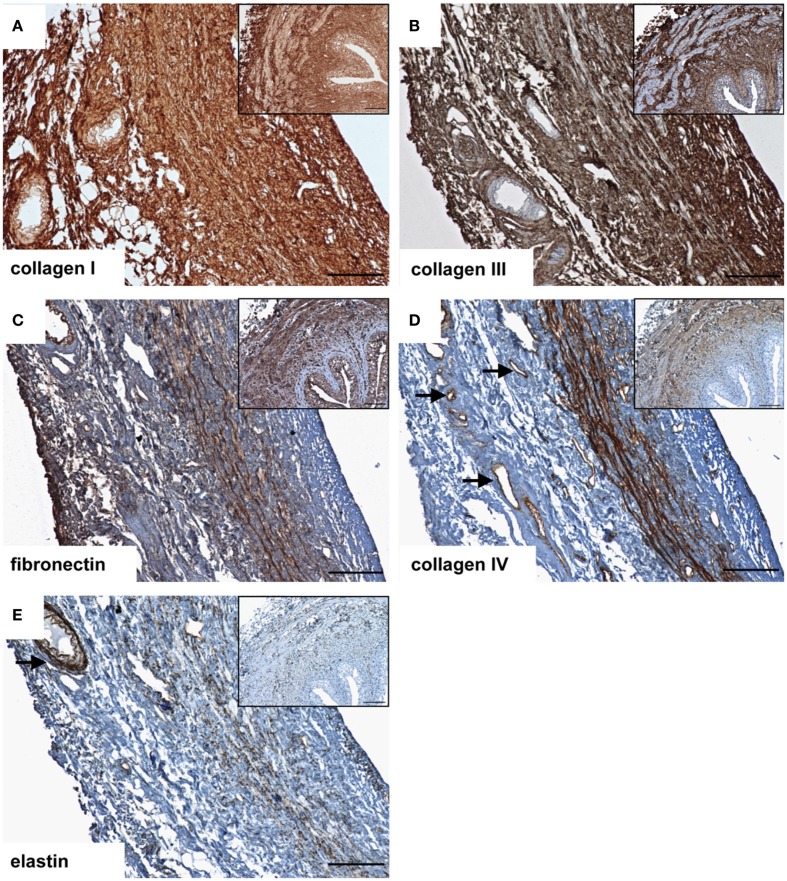
**Immunohistochemical DAB-staining of decellular porcine ureteral scaffolds for collagen I (A), collagen III (B), fibronectin (C), collagen IV (D), and elastin (E) in comparison to natural tissue (small pictures)**. Decellular ureteral scaffolds were shown to maintain native extracellular matrix composition. The arrows mark vessels. Bar = 100 μm.

After decellularization, the ECM composition is similar to native ureteral tissue. Azan staining of natural (Figure 1A) and decellular ureteral scaffolds (Figure 1B) revealed morphologically intact structures, ideal matrix geometry, and no remaining cellular structures. Histological analysis after lyophilization also showed preserved morphological structures and matrix geometry (Figure 1C) similar to the native and decellular conditions. After decellularization, collagen I (Figure 2A), collagen III (Figure 2B), and fibronectin (Figure 2C) could be observed in large amounts in all tissue areas similar to native conditions (small pictures, Figures 2A–C). The vessels expressed collagen IV (Figure 2D) and elastin, which are also located in tunica muscularis (Figure 2E).

### DNA quantification

**Figure 3:**
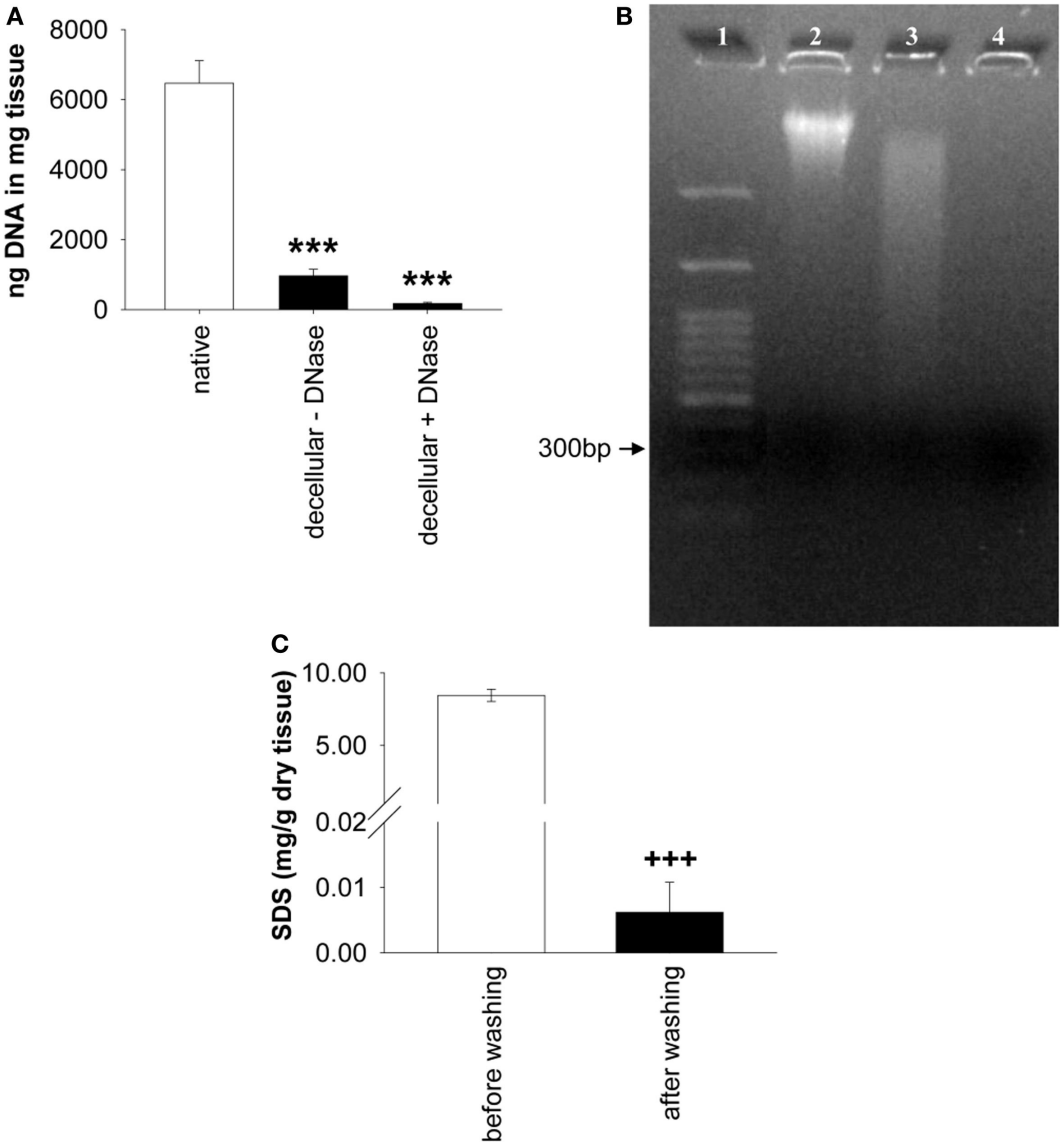
**(A) Shows the comparison of remnant DNA in decellular ureteral tissue with and without DNA digestion (both *n* = 9)**. Significant differences between both groups were not found. Native, untreated ureteral tissue served as control (*n* = 9). In **(B)**, gel electrophoresis of native ureter sample (2) and corresponding decellular scaffold without (3) and with DNase (4) is shown. Lane numbers indicate the respective sample. **(C)** shows the analysis of residual SDS before and after washing in distilled water (*n* = 12). Decellular ureteral scaffolds showed high SDS-concentrations (8.45 ± 0.43 mg/g dry tissue) before washing compared to non-toxic SDS-concentration after washing with distilled water (0.01 ± 0.01 mg/g dry tissue). Washed scaffold pieces show residual SDS-concentrations of 0.07 ± 0.05% compared to scaffolds before washing (*P* < 0.001; not shown). ***P < 0.001 vs. native group. ^+++^*P* < 0.001 vs. decellular ureteral scaffolds before washing.

The analysis of the DNA content revealed significant differences among native ureters, decellular ureters, and decellular ureters + DNA digestion [*P* < 0.001; each *n* = 9]. The DNA content in decellular scaffolds was significantly lower (85.01 ± 3.1% = 966.1 ± 188.2 ng/mg tissue; *P* < 0.001) compared to that in native ureter samples (100% = 6,468.11 ± 646.9 ng/mg tissue). An additional DNA digestion further reduced the amount of the DNA to 97.32 ± 0.7% (173.28 ± 36.6 ng/mg tissue) compared to native ureter samples (*P* < 0.001; Figure 3A. Differences between decellular scaffolds and decellular scaffolds+ DNA digestion did not reach a level of significance (*P* = 0.31). The percentage of remaining DNA after both procedures was decreased by about 91.2% compared with native ureteral tissue (*P* < 0.001). Qualitative analysis by gel electrophoresis showed intact DNA bands in the native samples with a size larger than 3,000 base pairs. Decellularization caused a gross but incomplete removal of this band, accompanied by a visible DNA-smear. When treating the decellular scaffolds with DNase, the smear was grossly removed (Figure 3B).

### Residual SDS

A significant effect of the washing duration on the SDS-concentration was observed, resulting in a 99.93% decrease, compared to residual SDS before washing. The concentration of SDS within the scaffolds after washing in distilled water was 0.006 ± 0.01 mg/g dry tissue vs. 8.449 ± 0.43 mg/g dry tissue in scaffolds before washing ([F_(1,11)_ = 391.89, *P* < 0.001], each *n* = 12, Figure 3C).

### Reseeding decellular ureteral scaffolds

**Figure 4:**
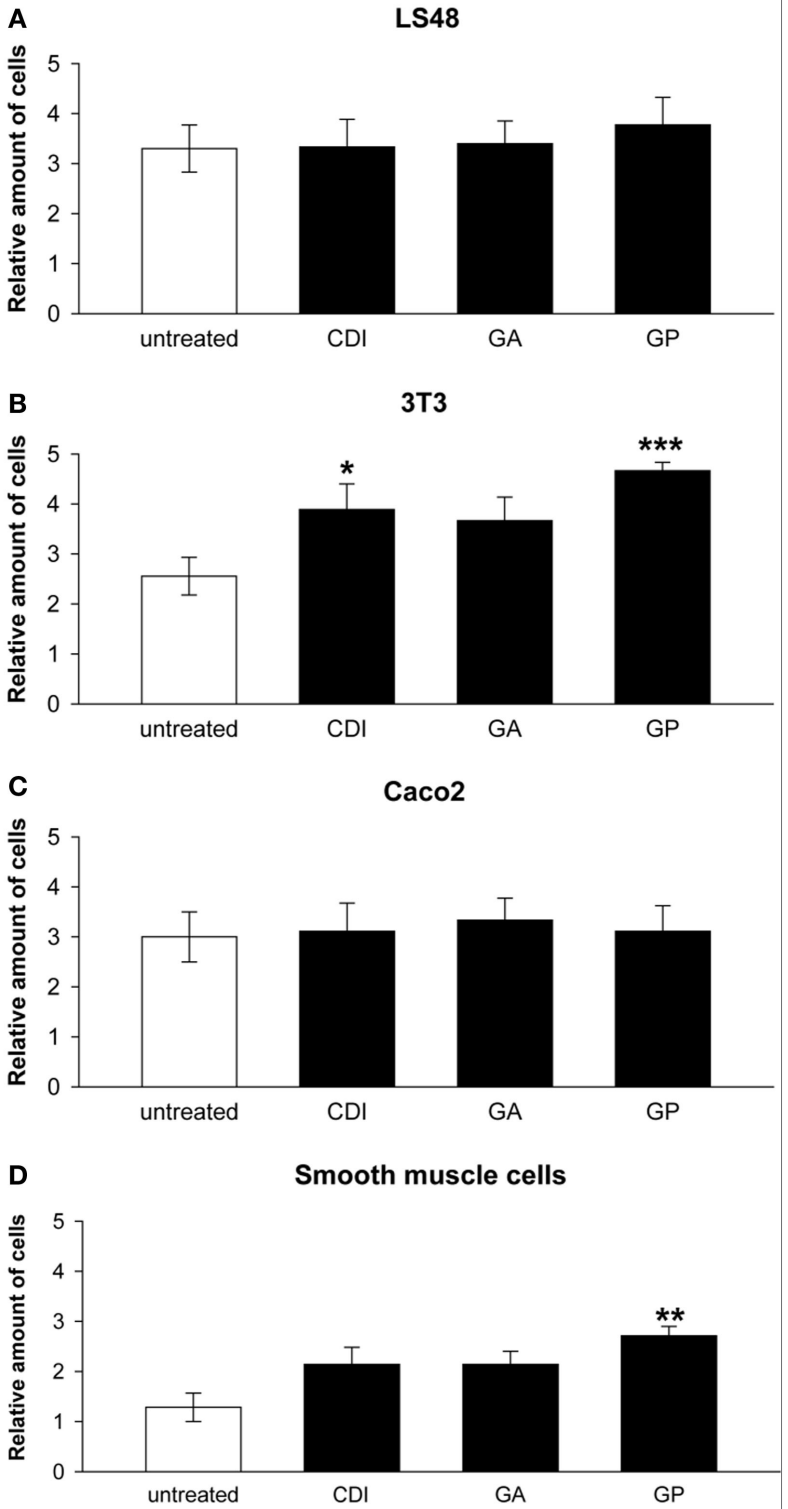
**Reseeding of crosslinked or chemical untreated ureteral scaffolds with LS48 (A), 3T3 (B), aco2 (C), and smooth muscle cells (D)**. Cells are able to grow on chemically untreated and crosslinked scaffolds after incubation for 2 weeks. Effects of crosslinking on relative amount of cells could not be detected under seeding with LS48 [**(A)**, *n* = 9] and Caco2 [**(C)**, *n* = 9] cells. However, 3T3 cells show an increased infiltrating rate into CDI and GP crosslinked scaffolds, compared to the untreated group [**(B)**, *n* = 9]. Furthermore, crosslinking with GP caused an increase of smooth muscle cells, grown on surface and infiltrate scaffolds [**(D)**, *n* = 7]. Data were calculated based on the scoring by two blinded observers: 1 = no cells; 2 = in total <20 cells; 3 = partially 1 cell layer, in total <50 cells, 4 = 1 cell layer around the scaffold; 5 = cells are arranged in multilayer, with matrix-infiltration. Untreated, untreated decellular ureteral tissue; GP, genipin; GA, glutaraldehyde; CDI, carbodiimide. *P < 0.05, **P < 0.01, ***P < 0.001 vs. decellular ureteral scaffolds.

**Figure 5:**
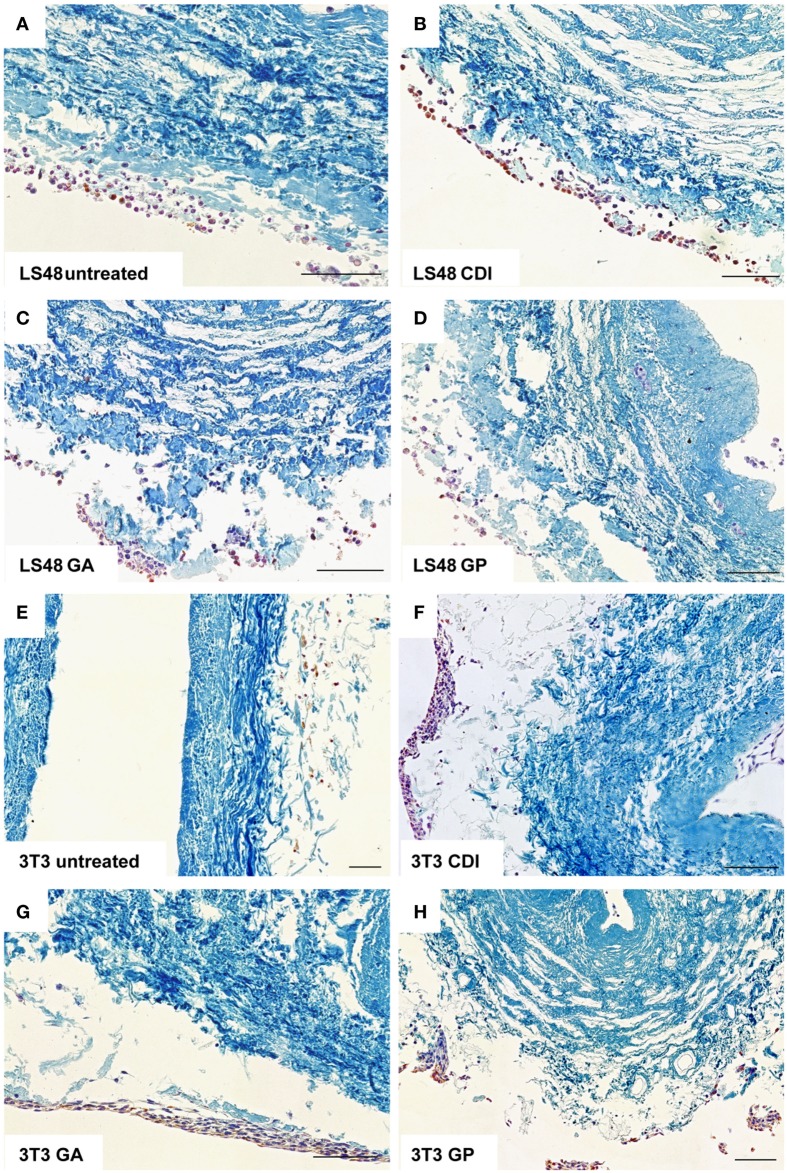
**Reseeding of crosslinked or chemical untreated ureteral scaffolds with LS48 and 3T3 cells**. LS48 cells grew sporadically on scaffolds and showed small clusters. However, crosslinking showed no effect on capability of cells to grow on scaffolds **(A–D)**. Furthermore, 3T3 cells prefer crosslinked scaffolds **(E–H)**. Multilayer formations and infiltration was predominant in CDI **(F)** and GP **(H)** crosslinked scaffolds, whereas only few cells were detectable in untreated scaffold group.

**Figure 6:**
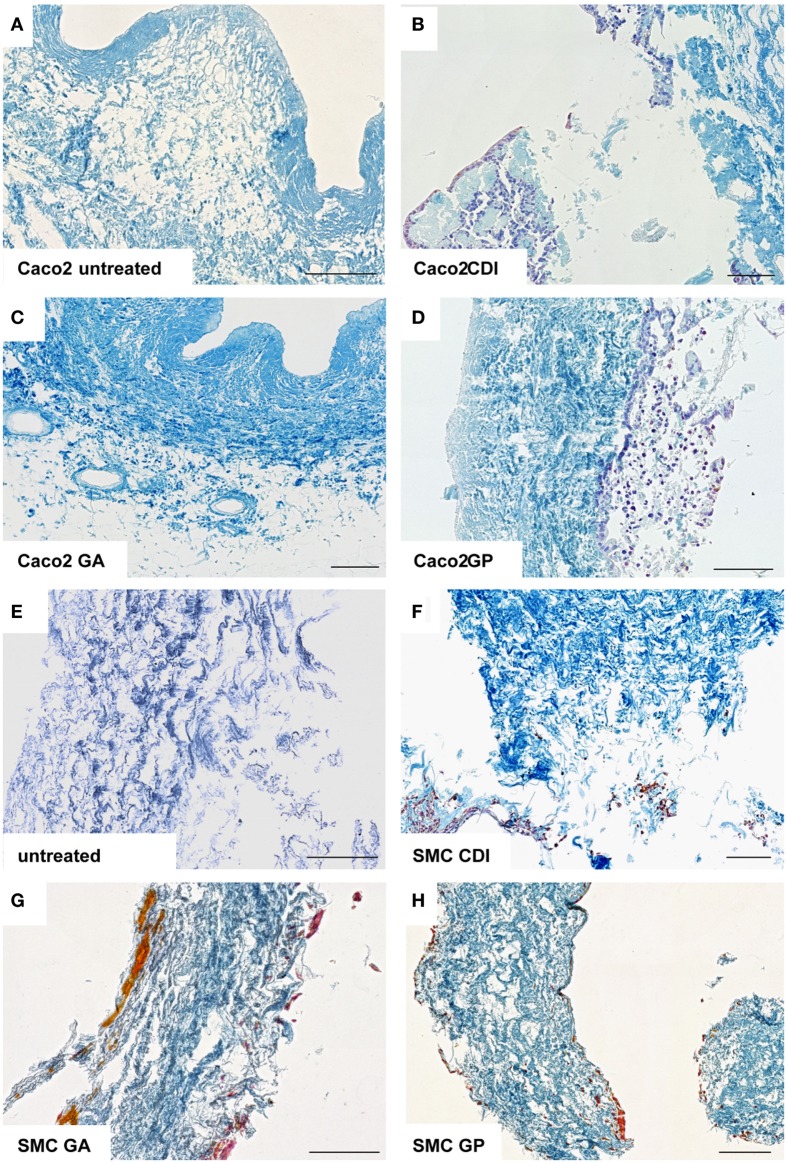
**Reseeding of crosslinked or chemically untreated ureteral scaffolds with Caco2 and rat smooth muscle cells**. Only a low rate of Caco2 cells was detectable in untreated **(A)** and GA crosslinked **(C)** scaffolds. After CDI **(B)** and GP **(D)** crosslinking, Caco2 cells showed multilayer formation and scaffold infiltration. Smooth muscle cells also preferred crosslinked scaffolds **(E–H)**, whereas cell-cluster and infiltration were observed. Untreated, untreated decellular ureteral tissue; GP, genipin; GA, glutaraldehyde; CDI, carbodiimide. Bar = 100 μm.

After incubation for 2 weeks, the ureteral scaffolds were increasingly infiltrated by tested cell lines (Figures 4 and 5). LS48 cells were uniformly distributed at the scaffold surface (Figures 5A–D). No significant effects of crosslinking were observed on cell growth (Figure 4A). 3T3 cells showed a higher infiltration after crosslinking with CDI or GP ([F_(3,24)_ = 6.84, *P* = 0.002]; CDI: *P* < 0.05, GP: *P* = 0.001; Figure 4B). Furthermore, multilayer formation (GA, CDI, GP) and moderate scaffold infiltration (CDI, GP) were detected in crosslinked scaffolds, whereas only sporadic cells could be detected in the untreated scaffold group (Figures 5E–H). After incubation with Caco2 cells, the relative amount of cells did not differ between any of the groups (Figure 4C). However, scaffold infiltration and multilayer formation were predominant after crosslinking with GP or CDI (Figures 6A–D). Furthermore, SMC showed a preference for crosslinked scaffolds (Figures 6E–H), whereas the highest infiltration was observed after crosslinking with GP ([F_(3,18)_ = 5.32, *P* < 0.008]; *P* < 0.01; Figure 4D). However, reseeding of lyophilized scaffolds failed; neither cell lines nor native cells were grown on dried scaffolds (data not shown). Interestingly, lyophilized implants were degraded by cell lines within 2 weeks.

### Response to implanted decellular ureteral scaffolds

**Figure 7:**
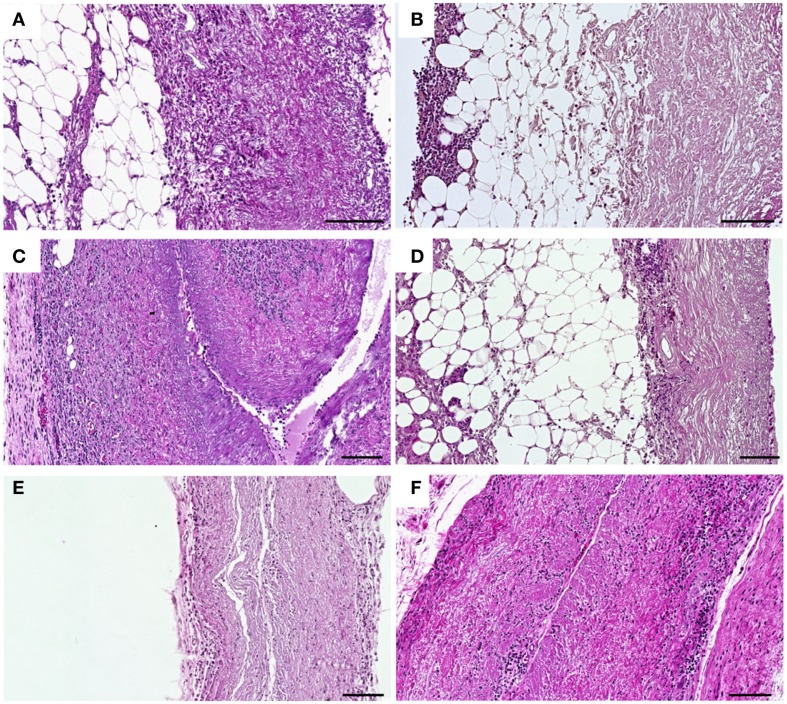
**Histological analysis of HE-stained subcutaneous implanted untreated (A,C,E) and CDI crosslinked scaffolds (B,D,F) explanted at days 1, 9, and 30 post-operative**. At day 1 post implantation, cellular infiltration with granulocytes, fibroblasts, and macrophages could be observed in untreated scaffolds **(A)**, whereas in CDI crosslinked scaffolds **(B)** only a cellular layer was detectable at the periphery. At day 9 post implantation, a notable increase of infiltrating cells into untreated scaffolds **(C)** was detected as a sign of encapsulation. CDI crosslinked **(D)** scaffolds showed a mild cellular infiltration with granulocytes, fibroblasts, and macrophages. At day 30 post implantation, in contrast to the untreated scaffold group **(E)**, CDI crosslinked scaffolds **(F)** showed only a marginal cellular infiltration by granulocytes, fibroblasts, and macrophages. Untreated scaffolds were completely infiltrated by cells and largely degraded. Detailed cellular analysis is displayed in Figure 8. Bar = 100 μm.

The cell infiltration in untreated (Figures 7A,C,E) and crosslinked scaffolds (Figures 7B,D,F) was analyzed histologically after explantation at days 1, 9, and 30 post implantation. At day 1 after implantation, a cellular infiltration directed from the periphery to the center of the respective tissue was observed in untreated scaffolds (Figure 7A). After crosslinking with CDI (Figure 7B), a cellular layer was detectable at the periphery and only a few number of cells infiltrated the central parts of the scaffolds. At day 9 post implantation, a decrease of cell infiltration was observed in crosslinked scaffolds (Figure 7D) compared to that in untreated scaffolds (Figure 7C). At day 30 post implantation, the untreated implants were largely degraded and completely infiltrated with cells (Figure 7E). In the CDI crosslinked group only an immaterial cellular infiltration could be detected (Figure 7F). In all crosslinked groups (GP, GA, CDI, BP), the cell infiltration was lower compared to the untreated scaffold group but without significant differences among the groups.

**Figure 8:**
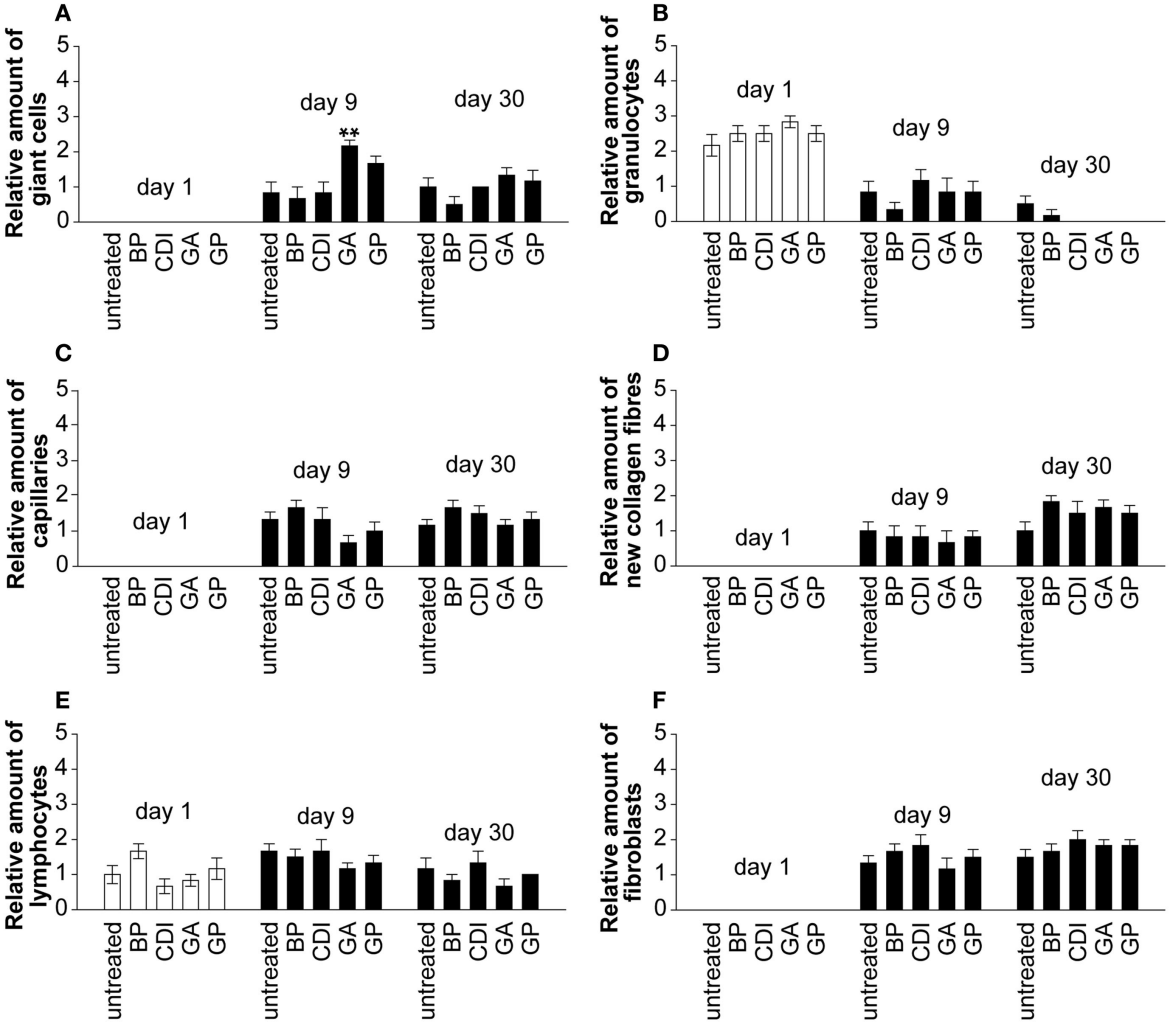
**Histological analysis of the degree of scaffold infiltration by giant cells (A), granulocytes (B), capillaries (C), collagen fibers (D), lymphocytes (E), and fibroblasts (F) at days 1, 9, and 30 post implantation**. In all implants, an increase of infiltrating giant cells, lymphocytes, fibroblasts, capillaries, and collagen fibers could be detected over time. Furthermore, a decrease of infiltration by granulocytes could be observed in all implants at day 30 compared to day 1 post implantation. A blinded pathologist evaluated the presence of cells (granulocytes, lymphocytes, giant cells, and fibroblasts), capillaries and new collagen fibers and generated a semiquantitative score in repeat determination (0 = negative, 1 = mild, 2 = moderate, 3 = serve; *n* = 6/group and day; five microscopic fields per slide and rat; each 5 mm^2^). Untreated, untreated decellular ureteral tissue; BP, bovine pericardium (St. Jude, USA); GP, genipin; GA, glutaraldehyde; CDI, carbodiimide. **P < 0.01 vs. untreated decellular ureteral tissue.

Only the GA crosslinked group showed a significant increase of giant cells compared to untreated group at day 9 post implantation (Figure 8A, *P* < 0.01). At day 1 post implantation, granulocytes were present in each group. Their number decreased and diminished toward day 30 post implantation in most groups, excepting the untreated and BP group (Figure 8B). Neovascularization (Figure 8C), new collagen fibers (Figure 8D), and fibroblasts (Figure 8F) were present in all groups without significant differences. At day 1 after implantation, lymphocytes were present in each group and did not differ to day 9 or 30 post-operative (Figure 8E).

**Figure 9:**
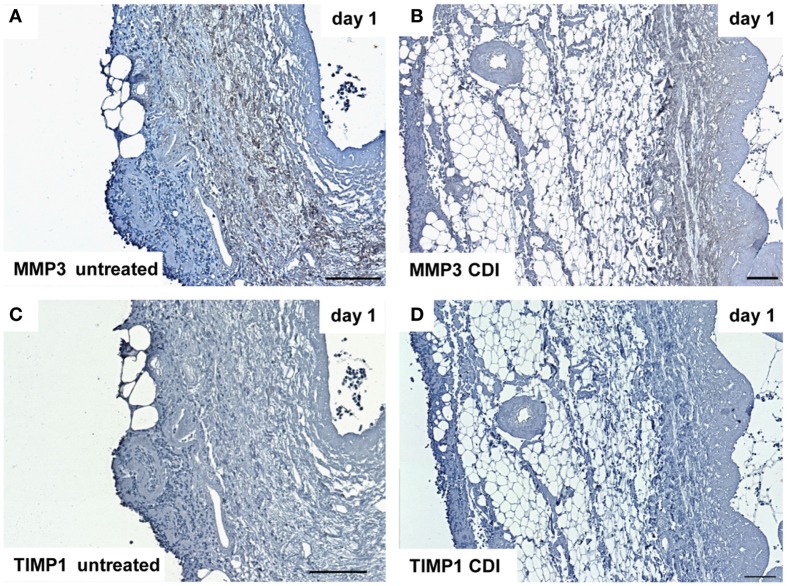
**Immunohistochemical DAB-staining of decellular CDI crosslinked implants for MMP3 and TIMP1 in comparison to chemical untreated scaffolds after 1, 9, and 30 days *in vivo* (*n* = 6/day/group)**. One day post implantation, MMP3 were detectable in both untreated **(A)** and CDI crosslinked **(B)** scaffolds, whereas CDI crosslinked scaffolds showed a weaker intensity. However, scaffolds of both groups were negative for TIMP1 [untreated: **(C)**, CDI: **(D)**].

**Figure 10:**
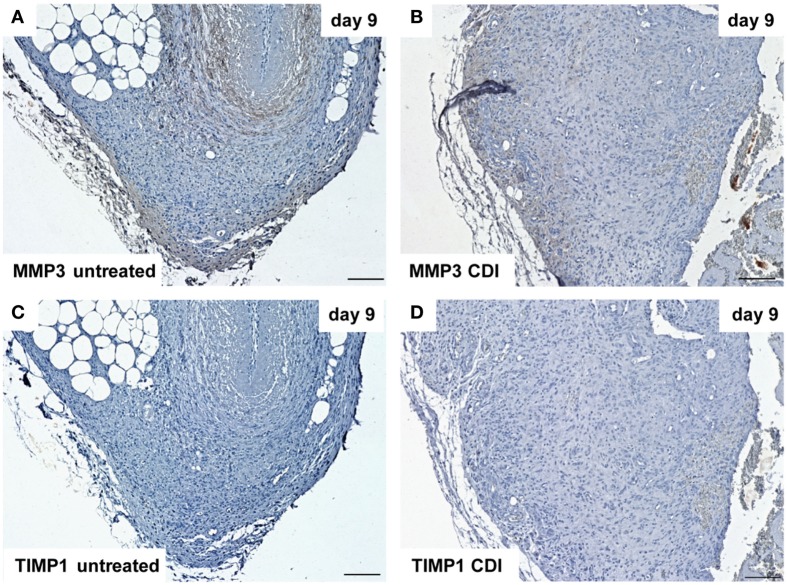
**Immunohistochemical DAB-staining of decellular CDI crosslinked implants for MMP3 and TIMP1 in comparison to chemical untreated scaffolds after 1, 9, and 30 days *in vivo* (*n* = 6/day/group)**. Nine days post-implantation, the rate of MMP3 [untreated: **(A)**, CDI: **(B)**] reactivity increased compared to post-operative day 1. TIMP1 reactivity could not be detected in both untreated **(C)** and crosslinked **(D)** scaffolds.

**Figure 11:**
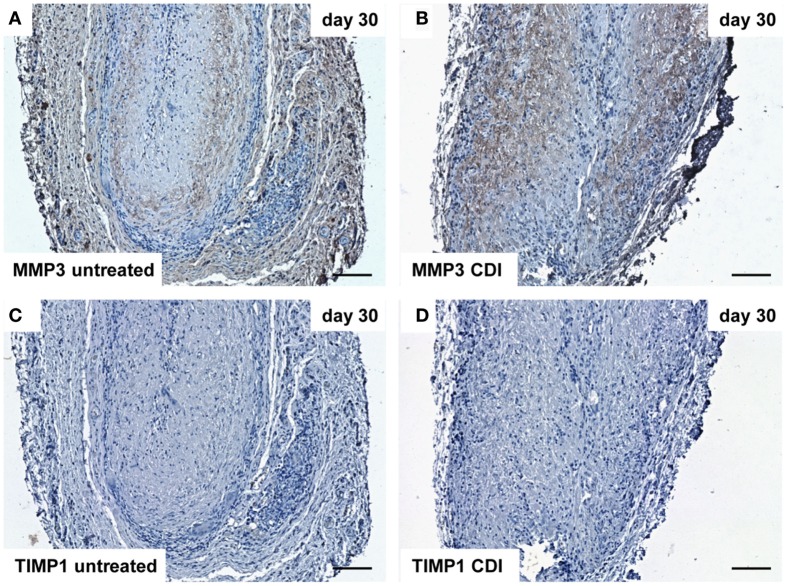
**Immunohistochemical DAB-staining of decellular CDI crosslinked implants for MMP3 and TIMP1 in comparison to chemical untreated scaffolds after 1, 9, and 30 days *in vivo* (*n* = 6/day/group)**. At day 30 post implantation, MMP3 could be detected in both groups [untreated: **(A)**, CDI: **(B)**], whereas TIMP1 was not detectable in both untreated **(C)** and CDI crosslinked **(D)** scaffolds. Bar = 100 μm.

At day 1 after implantation, MMP3 was activated in all groups (untreated: Figure 9A, crosslinked: Figure 9B) and increased to day 9 (untreated: Figure 10A, crosslinked: Figure 10B), whereas the reactivity was weaker in crosslinked than in untreated scaffolds. At day 30, MMP3 was detectable in both untreated (Figure 11A) and crosslinked (Figure 11B) scaffolds with same intensity. Scaffolds of all groups were negative for TIMP1 at all time-points (Figures 9C,D; Figures 10C,D; Figures 11C,D).

**Figure 12:**
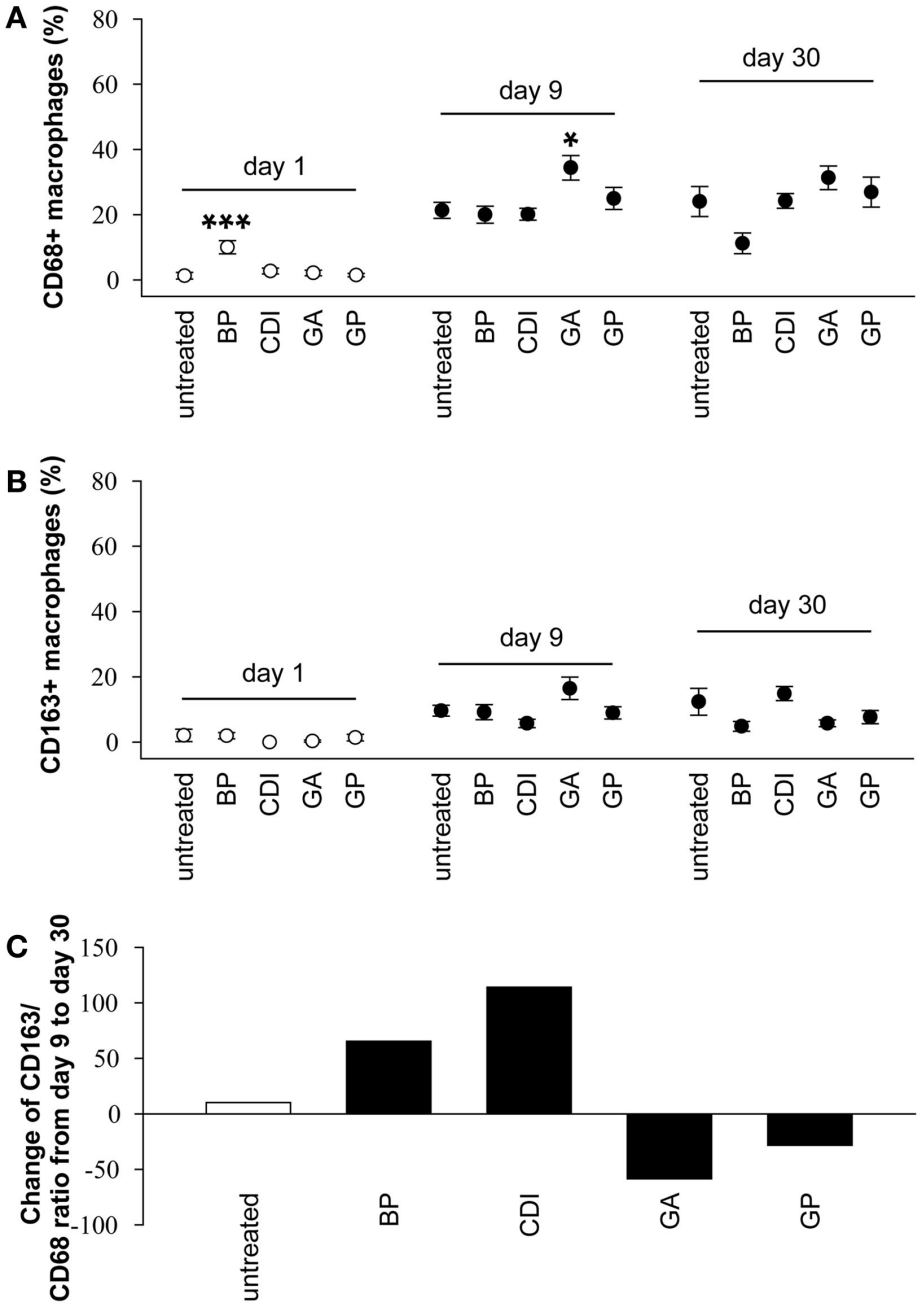
**Immunohistochemical analysis of the degree of scaffold infiltration by CD68 + (A) and CD163 + (B) macrophages after 1, 9, and 30 days, as well as the change of the CD163/CD68 ratio from day 9 to day 30 (C)**. An increase of CD68 + and CD163 + macrophages could be observed in all groups from day 1 to day 9, whereas the amount remains constant to day 30 post implantation. In addition to the standard group, untreated and CDI-crosslinked scaffolds showed a macrophage M2 phenotype switch, indicated by a positive CD163/CD68 ratio at day 30 compared to ratio at day 9. All data were represented as ratios of specific cells/total cells ± SEM. Five microscopic fields (magnification × 000) of one slide per rat were analyzed (*n* = 6/group and day). An average of 54 ± 1.59 total cells per microscopic field was counted to generate the ratio of cells/total cells. Untreated, untreated scaffold; BP, bovine pericardium (St. Jude, USA); GP, genipin; GA, glutaraldehyde; CDI, carbodiimide. *P < 0.05, **P < 0.01 vs. untreated decellular ureteral tissue.

A detailed macrophage analysis showed only a few number of CD163 positive cells at the periphery of BP and CDI crosslinked samples at day 1 after implantation. Furthermore, the number of CD68 positive cells was higher in the BP group compared to that in untreated scaffolds ([F_(4,25)_ = 9.80, *P* < 0.001]; *P* < 0.001, Figure 12A). Scaffold analysis at day 9 after implantation revealed a significant increase of CD68 positive macrophages after crosslinking with GA compared to the untreated group ([F_(4,25)_ = 4.40, *P* = 0.008]; *P* < 0.05). At day 30 post-operative, there were no significant effects of crosslinking on the number of CD68 and CD163 positive cells (Figure 12B). Additionally, in most groups, the amount of the anti-inflammatory, pro-remodeling macrophage M2 phenotype increased from day 9 to day 30, indicated by a positive CD163/CD68 ratio (untreated: +10.05%; BP: +65.37%; CDI + 114.03%), whereas it decreased in the GP (−28.31%) and GA (−58.48%) groups (Figure 12C).

## Discussion

Different surgical techniques were employed for ureter reconstruction such as Boari flap, psoas hitch, downward mobilization of the kidney, and replacement with ileal or bladder tissue. These reconstructing techniques are accompanied by several complications (i.e., excessive formation of mucus, ureteral stenosis, infection, renal, chronic renal failure, and metabolic disturbances or urolithiasis) (Corvin et al., 2004; Schlote et al., 2004; Wolff et al., 2011). Therefore, the development of a biocompatible ureteral implant would be of high clinical interest. Different types of scaffold materials such as hydrogels, synthetic, or natural scaffolds have already been studied in tissue engineering (Dahms et al., 1997). Partial or total ureteral replacement by synthetic materials such as teflon (Ulm and Krauss, 1960) or dacron (Block et al., 1977) was examined. Unfortunately, these materials showed a lack of biocompatibility, peristalsis, and moderate incrustation. Biological ECM scaffolds possess intact structural proteins and growth factors that reduce inflammatory responses (Badylak, 2004; Chang et al., 2005; Badylak and Gilbert, 2008). Nevertheless, decellular scaffolds have several advantages over synthetic implants. The scaffold materials can be easily obtained from different species. The cellular components can be removed from the tissue/organ by a broad range of various methods (Gilbert et al., 2006; Koch et al., 2012), resulting in decellular ECM. Structural proteins of the ECM remain intact and ECM scaffolds have a three-dimensional structure that facilitates host cell ingrowth (Badylak, 2004; Koch et al., 2012). Due to removing the DNA and cellular components, the antigenic binding sites were strongly reduced. Therefore, one might assume that decellular scaffolds do not provoke a chronic rejection reaction after implantation, even if originating from another species. In 1997, first preliminary experiments with decellular matrixes showed promising results concerning the ingrowth of urothelium, SMC and nerve cells (Dahms et al., 1997). However, complications such as ureteral occlusion and finally hydronephrosis were also described (Sofer et al., 2002). In addition, previously published data suggest that the host response to the ECM scaffolds was strongly dependent on the species and chemical pre-treatment (Sung et al., 1999a,b; Chang et al., 2002, 2005; Liang et al., 2004; Badylak and Gilbert, 2008; Cao and Xu, 2008; Everaerts et al., 2008; Koch et al., 2012). Therefore, currently published data are not universally consistent. Thus, it was first essential to test the morphological characteristics and the biocompatibility of the ureteral scaffolds.

To the best of our knowledge, we are the first to describe the decellularization of porcine ureters and investigated the influence of different crosslinking agents on cellular reseeding *in vitro* and on the inflammatory response *in vivo* in one study.

### Ureter decellularization and matrix composition

According to our hypothesis, porcine ureters can be easily decellularized. After decellularization, histological analysis revealed morphological intact structures, optimal matrix geometry and no remaining cellular structures. Lyophilized ureters showed intact morphological structures, whereas collagen I, III, IV, fibronectin, and elastin could be observed in amounts similar to natural or decellular ureters.

### Success of *In Vitro* reseeding depends on crosslinker

*In vitro* data showed a considerable ingrowth of different cell lines (LS48, 3T3, Caco2) and SMC), depending on crosslinking. Highest infiltration was offered by the crosslinkers CDI and GP compared to other crosslinkers and chemically untreated scaffolds. These findings reflect not only the positive effects of crosslinking but also the effectiveness of washing out residual cytotoxic SDS concentrations (remove of 99.93% SDS). In contrast to crosslinking with GP or CDI, the infiltration of cells into GA crosslinked scaffolds was comparable to untreated scaffolds *in vitro* as a sign of non-optimal growth conditions. After GP crosslink, an increase of ingrown cells was described compared to GA crosslink (Sung et al., 1999b; Koch et al., 2012), whereas signs of calcification were not detected in GP fixed tissue (Sung et al., 1999a). In the present study, we found a significantly increased ingrowth and multilayer formation of 3T3 and SMC after GP crosslinking compared to that in untreated scaffolds *in vitro*. In comparison to untreated scaffolds, CDI crosslinked scaffolds showed an increase of infiltrating 3T3-cells and multilayer formation as a sign of optimal environment comparable to GP-treated group. In contrast to recent studies, cells did not grow on lyophilized ureters (Kim et al., 2012; Woon et al., 2012); this might indicate that molecular characteristics were changed during lyophilization. On the basis of the facts, lyophilization could not be an optimal alternative to store ureteral scaffolds at this time. However, it would be relevant to analyze molecular changes after lyophilization of crosslinked scaffolds in further studies.

### Inflammation, tissue resorption and remodeling processes depend on crosslinker *In Vivo*

In addition, it is fundamental to determine the host response after subcutaneous implantation of different crosslinked ureteral scaffolds. Since the scaffolds were decellular and morphologically intact, we could show in a subcutaneous rat model with decellular esophagus scaffolds that chronic rejection will not occur and implants are well tolerated (Koch et al., 2012). The present work supplements the biocompatible characteristics of acellular scaffolds by reseeding experiments, DNA analysis, and an SDS assay. At days 9 and 30 post implantation, signs of inflammation such as encapsulation and scaffold infiltration by macrophages and fibroblasts were observed and consistent with our expectations. We do not assume that DNA remnants of the scaffolds were the cause of inflammatory reactions in our experiments. After DNA extraction, we could not detect large remnant DNA fragments. Furthermore, we could show a reduction in remnant DNA of 91.2% compared to that of native ureters. In most biological material, remaining DNA consisted of fragments <300 bp, whereas DNA in our experiments almost disappeared. The small amount of remnant DNA is subject to fast enzymatic degradation *in vivo* (Badylak and Gilbert, 2008). It is more plausible, that free amino (–NH_2_), carboxyl (–COOH) and hydroxyl (–OH) groups of collagen may be responsible for the immunological reactions (Sung et al., 1999b; Liang et al., 2004; Chang et al., 2005; Cao and Xu, 2008; Koch et al., 2012). These free groups can be bound by chemical crosslinking, which prevents the development of antigenic properties (Ye et al., 1996; Khor, 1997). Another side effect of crosslinking is an increase in mechanical stability. However, some crosslinking agents (e.g., GA) are toxic or promote the calcification of scaffolds *in vivo* (Khor, 1997; Sung et al., 1999a; Chang et al., 2002; Everaerts et al., 2008; Koch et al., 2012). In the present study, the crosslinkers GA, GP, and CDI were selected in accordance with descriptions of the biocompatibility in the recent literature (Khor, 1997; Sung et al., 1999b; Liang et al., 2004; Chang et al., 2005; Cao and Xu, 2008; Koch et al., 2012; Jeong et al., 2013).

Glutaraldehyde is an aggressive, indiscriminant crosslinking reagent that is commonly used in commercially available tissues (Badylak and Gilbert, 2008). GA crosslinked tissue exhibits a stabilized collagen matrix and decreased immunological reaction (Jeong et al., 2013; Awang et al., 2014). However, we observed that the immunological response (giant cells, CD68+ macrophages) in GA crosslinked ureteral scaffolds was higher compared to that in chemically untreated scaffolds. Furthermore, the polarization of macrophages is important to the remodeling outcome. M1-activated macrophages produce inflammatory cytokines, which promote active inflammation and were associated with rejection reactions. In contrast, M2-activated macrophages (CD163+) are able to facilitate tissue repair and constructive remodeling (Mantovani et al., 2005; Brown et al., 2009; Chin et al., 2011). Macrophages are able to change their polarization in response to local stimuli during the process of wound healing (Mantovani et al., 2004; Stout and Suttles, 2005; Martinez et al., 2008; Kushiyama et al., 2011). The recognition of the predominant phenotype of macrophages provides an indication of scaffold rejection, inflammation, or acceptance after implantation. Interestingly, in contrast to the untreated, BP, and CDI groups, the CD163/CD68 ratio was decreased after GA treatment at day 30 post implantation. This might indicate a switch to a pro-inflammatory and destructive M1 macrophage phenotype after GA treatment. Furthermore, cellular toxicity and cytotoxic T-cell activation have been described after incomplete suppression of immunological actions of GA crosslinked scaffolds (Nishi et al., 1995; Chang et al., 2002; Kim et al., 2014; Manickam et al., 2014). On the other hand, cytotoxic effects of GA crosslinked scaffolds for host cells (fibrocytes, fibroblasts, and macrophages) were described (Huang et al., 1998). We did not observe any cytotoxic effect of GA crosslinked ureteral scaffolds on host fibroblasts or macrophages *in vivo*. In addition, further disadvantages of GA (e.g., scaffold calcification and depolymerization of GA crosslinks) have been reported (Khor, 1997; Schoen and Levy, 2005; Liu et al., 2014). The data suggest that GA was not an optimal crosslinker for constructive remodeling in the present study (e.g., giant cell and CD68 + macrophage infiltration *in vivo*, poor biocompatibility *in vitro*), which corresponds with other disadvantages described in the literature (Nishi et al., 1995; Huang et al., 1998; Koch et al., 2012).

Genipin has been widely used as a natural crosslinker as a substitute for chemical crosslinkers. It reacts with collagen amino groups and is approximately 10,000 times less cytotoxic than GA (Sung et al., 1999b). The stable GP crosslinked products protect against inflammation, degradation, react antiphlogistically and resulted in faster tissue regeneration compared to GA (Liang et al., 2004; Koo et al., 2006; Li et al., 2012). Furthermore, a significantly decreased inflammatory response compared to untreated and GA crosslinked scaffolds is described after implantation of GP and GA crosslinked decellular bovine pericardia (Chang et al., 2002). However, in our subcutaneous rat model, we could show that macrophages were present in all scaffolds (untreated and crosslinked) at days 1, 9, and 30 post implantation. In contrast to previous studies (Koch et al., 2012), we also detected a M1 macrophage phenotype switch after GP crosslinking comparable to the GA group, indicating pro-inflammatory and destructive processes. In this way, our data suggest that GP was not the optimal crosslinker for constructive remodeling.

The alternative crosslinker, CDI, activates carboxyl groups for spontaneous reaction with primary amines of aspartic acid and glutamic acid residues of collagen, generating the crosslink (Khor, 1997). Furthermore, recent literature showed that tissue quality was improved, calcification was decreased and tissues offered a good biocompatibility (Khor, 1997). Based on these facts, it is not remarkable that CDI was also used in commercially available tissue products (Badylak and Gilbert, 2008). However, it was reported that CDI crosslinking caused a decrease in elasticity and mechanical toughness (Rafat et al., 2008). In this study, we did not analyze the mechanical properties, but we observed that CDI and GP fixed scaffolds exhibited only a moderate degradation. However, a predominant M1 macrophage phenotype was observed after implantation of CDI crosslinked esophagus scaffolds in a recent study (Koch et al., 2012). Interestingly, we detected a remarkable M2 macrophage phenotype switch after CDI crosslinking *in vivo*, which is known to be associated with constructive remodeling and tissue repair (Khor, 1997; Chang et al., 2002; Koch et al., 2012). Furthermore, the presence of pro-remodeling macrophages and fibroblasts might suggest host repair and constructive remodeling (Chin et al., 2011). Furthermore, the infiltration, survival, and living of the tissue-fibroblasts support the good biocompatibility of CDI scaffolds *in vivo*.

In addition, the activation of MMP3 and the inactivation of TIMP1 indicate remodeling and healthy processes and were also analyzed at each point of time. MMP3 degrades fibronectin, laminin, elastin, collagen (II, IV, IX, X, XI), and activates collagenase1 (Bullard et al., 1999; Shantha Kumara et al., 2014). MMP3 was synthesized by fibroblasts, activated macrophages and keratinocytes adjacent to sites of injury and was found in settings where active ECM remodeling occurs (Bullard et al., 1999). Furthermore, MMP3 can also activate other MMPs such as MMP1, MMP7, and MMP9, rendering MMP3 crucial in connective tissue remodeling (Ye et al., 1996). The enzyme is not only considered to be involved in wound repair but also in progression of atherosclerosis and tumor initiation (Shantha Kumara et al., 2014). In the present study, MMP3 was detectable in each group, whereas reactivity was decreased after crosslinking compared to untreated scaffolds, as a sign of increased stability, required for adequate constructive remodeling processes. The MMP3 reactivity after crosslinking (GA, GP, CDI) reached its maximum in the CDI group, at day 30 post-operative. TIMP1, a tissue inhibitor of metalloproteinases, is a glycoprotein that is expressed from several tissues of organisms, able to promote cell proliferation in a wide range of cell types, and might also have an anti-apoptotic function. In the present study, TIMP1 was not detected at any point in time.

## Conclusion

We reported on morphology, *in vitro* biocompatibility, and immune response of tissue-engineered decellular porcine ureteral scaffolds, treated with different crosslinking agents. CDI crosslinked scaffolds exhibited a crucial M2-macrophage phenotype switch, activated MMP3, and inactivated TIMP1 *in vivo*. The results suggested constructive remodeling processes and an improved integration of ureteral scaffolds into their surrounding tissue after implantation. These results were supported by *in vitro* results: a high infiltration of different cells was observed in CDI and GP scaffolds. The sum of the data suggests that CDI offered most benefits for crosslinking ECM scaffolds. The results of the present study help to develop a new biocompatible ureteral xenograft. However, biomechanical data are necessary to investigate a ureteral scaffold. In the future, we will design experiments to investigate biomechanical characteristics of decellularized tissue compared to native. Furthermore, studies such as large animal models should clarify the functionality of segmental CDI crosslinked ureteral scaffolds in the ureteral location. If this approach is successful, decellular ureteral scaffolds could be an important therapeutic tool for a wide range of applications (e.g., malignant tumor, ureteral stenosis, ureteral atresia, etc.).

## Conflict of Interest Statement

The authors declare that the research was conducted in the absence of any commercial or financial relationships that could be construed as a potential conflict of interest.
